# Reproductive Outcomes and Endocrine Profile in Artificially Inseminated versus Embryo Transferred Cows

**DOI:** 10.3390/ani10081359

**Published:** 2020-08-06

**Authors:** Jordana S. Lopes, Estefanía Alcázar-Triviño, Cristina Soriano-Úbeda, Meriem Hamdi, Sebastian Cánovas, Dimitrios Rizos, Pilar Coy

**Affiliations:** 1 Physiology of Reproduction Group, Departamento de Fisiología, Facultad de Veterinaria, Universidad de Murcia, Campus Mare Nostrum, 30100 Murcia, Spain; jordanaluisa.portugals@um.es (J.S.L.); cmsu1@um.es (C.S.-Ú.); scber@um.es (S.C.); 2 Institute for Biomedical Research of Murcia, IMIB-Arrixaca, 30120 Murcia, Spain; 3 El Barranquillo S.L., Torre Pacheco, 30700 Murcia, Spain; estefania.alcazar@elbarranquillo.es; 4 Department of Animal Reproduction, National Institute for Agriculture and Food Research and Technology (INIA), 28040 Madrid, Spain; mhamdi9186@hotmail.com (M.H.); drizos@inia.es (D.R.)

**Keywords:** embryo transfer, reproductive fluids, pregnancy, vitrification, calving

## Abstract

**Simple Summary:**

Bovine embryos are nowadays produced in laboratories, frozen and transferred to other cows. However, the percentage of pregnancies obtained after these transfers as well as difficulties found during labor, especially due to increased size of calves, are a matter of great concern. One of the possible explanations for these problems relies on the embryo being produced in in vitro conditions (laboratory settings), more specifically the culture medium (liquid) used to develop these embryos. In an attempt to better mimic what happens naturally, female reproductive liquids (from oviducts and uterus) were used as a supplement to the culture of the embryos. As controls, embryos produced using the standard protocol in the laboratory were produced, as well as embryos derived from artificial insemination of cows (in vivo). An evaluation on the pregnancy rates, how the hormonal profile of the recipients changed during pregnancy, difficulties during parturitions, and phenotype of calves were recorded. Results showed that all the groups were very similar, but many differences were noted on the hormonal profiles during pregnancy. In conclusion, all systems provided safe production of calves, but long-term analysis of these calves is necessary to understand the future impact of the laboratory protocols.

**Abstract:**

The increasing use of in vitro embryo production (IVP) followed by embryo transfer (ET), alongside with cryopreservation of embryos, has risen concerns regarding the possible altered pregnancy rates, calving or even neonatal mortality. One of the hypotheses for these alterations is the current culture conditions of the IVP. In an attempt to better mimic the physiological milieu, embryos were produced with female reproductive fluids (RF) as supplements to culture medium, and another group of embryos were supplemented with bovine serum albumin (BSA) as in vitro control. Embryos were cryopreserved and transferred while, in parallel, an in vivo control (artificial insemination, AI) with the same bull used for IVP was included. An overview on pregnancy rates, recipients’ hormonal levels, parturition, and resulting calves were recorded. Results show much similarity between groups in terms of pregnancy rates, gestation length and calves’ weight. Nonetheless, several differences on hormonal levels were noted between recipients carrying AI embryos especially when compared to BSA. Some calving issues and neonatal mortality were observed in both IVP groups. In conclusion, most of the parameters studied were similar between both types of IVP derived embryos and the in vivo-derived embryos, suggesting that the IVP technology used was efficient enough for the safe production of calves.

## 1. Introduction

In 2018, more than 1 million bovine embryos were produced worldwide from which the majority were in vitro produced (IVP) [1]. This represents a continuous growth of IVP-embryos globally despite the fact that IVP embryos have lower pregnancy rate than in vivo-derived embryos (IVD) [2,3]. Its success relies on the possibility of producing more embryos than in vivo, in a shorter amount of time and that the embryonic losses after implantation are no different from IVD, therefore, compensating the potential decrease in implantation rate [3,4]. In addition, the use of IVP-embryos is a better option during season of heat-stress [5] or in cases of repeat breeders [6].

Another technique on the rise is the cryopreservation of embryos, with more than 38% of 2018′s embryo transfers (ET) coming from frozen embryos [1]. The need for exchange of cattle genetics has popularized this embryo preservation technique, which has the added benefit to not have all the recipients synchronized at the time of fresh embryo production. Within these large numbers of ET of frozen embryos, almost half (>46%) were IVP-derived embryos, but only a very small percentage of these (<0.1%) were produced with oocytes from abattoir ovaries, the rest being obtained by ovum pick-up (OPU).

Although the increase in IVP is significant over the years, the quality of the embryos produced in vitro still remains inferior to IVD embryos [7,8,9]. This is a factor that is affected in all mammalian embryos, where culture conditions such as the type of medium, type of supplementations, gases concentration or even culture devices play a crucial role, determining the final yield and quality of the embryo [10]. Currently, research is leading us towards a more physiological approach to in vitro culture. Media supplementation is being updated, such as the addition of insulin-transferrin-selenium to culture medium [11] that increases the blastocyst yield, or the addition of reproductive fluids (RF) [12] that increases embryo quality by increasing their cryotolerance and protecting from oxydative stress. García-Martínez et al. [13] measured the levels of O_2_ within the different segments of the pig oviduct and uterus in vivo, and adapted the data obtained to the IVP, showing that decreasing the O_2_ concentration during IVF, though not affecting the IVF results directly, had a positive impact on blastocyst yield and quality. Also, a new device [14] named 3D oviduct-on-a-chip model was recently designed to promote a more accurate surface when culturing bovine embryos, allowing a proper fertilization of the oocytes and eliminating polyspermy and parthenogenic activation.

Several issues besides embryo quality have been pointed out regarding problems in IVP-pregnancies. Hasler [15] summarized them into four main points: (1) increased abortion rate; (2) reduced intensity of labour; (3) increased dystocia, birth weight, calf mortality and fetal abnormalities; and (4) higher percentage of male calves over female calves. However, in addition to these four issues, the fact that embryos are cryopreserved also affects their survival after warming [9,16,17], due to their high lipid content, as well as their implantation success, giving usually inferior pregnancy rates [15,18,19].

In order to further improve in vitro production and ultimately bovine-assisted reproductive technologies (ART), we designed the present study with the following specific objectives; (1) To find out whether culture media is improved by the addition of RF—acting closer to the physiological milieu—impacting the embryo implantation rate versus embryos produced in vitro with conventional culture media (BSA) or in vivo (AI); (2) To find out if hormonal levels in recipients, including P4, E2, AMH and cortisol, at day of ET and across pregnancy could help to predict ART-associated problems such as decrease implantation rate; and (3) To find out if RF-derived calves have more similarities with AI-derived calves, relative to conceptus size, gestation length, calving difficulty, and calf birth weight, than BSA-derived calves.

To fulfil our objectives, we used cow recipients allocated in three experimental groups, according to the origin of the embryo transferred: the first group received an in vitro produced embryo grown in culture media improved by supplementation with reproductive fluids (RF group); the second group received an embryo produced at the same time as those at the previous group but using conventional culture media, lacking reproductive fluids (BSA group); and the third group consisted of animals artificially inseminated with frozen-thawed semen from the same bull used in the two IVP groups (AI group).

## 2. Materials and Methods

All chemicals were purchased from Sigma-Aldrich Chemical Company (Madrid, Spain), unless otherwise indicated.

### 2.1. Ethics

The experimental work was submitted to evaluation by the CEEA (Comité Ético de Experimentación Animal) from University of Murcia. After approval, authorization from “Dirección General de Agricultura, Ganadería, Pesca y Acuicultura”, Región de Murcia- nr A13170706 was given to perform the animal experiments.

### 2.2. Oocyte Collection and In Vitro Maturation

Ovaries from crossbred beef cycling heifers and cows were collected at the slaughterhouse. The protocol has already been described elsewhere [12]. Briefly, follicles between 2–8 mm were aspirated and intact cumulus-oocyte complexes (COC) were selected for in vitro maturation (IVM). Groups of 50 COCs were cultured in maturation medium that consisted of TCM-199 supplemented with 10% fetal calf serum and 10 ng/mL epidermal growth factor for a period of 24 h at 38.5 °C, under 5%CO_2_ and high humidity.

### 2.3. In Vitro Fertilization

Commercially bought semen doses from one bull (Asturian Valley breed, ASEAVA, Asturias, Spain) were used for all the cycles of embryo production. Frozen semen straws were thawed in a water bath at 38 °C, and following the manufacturer’s instructions from Bovipure^®^ (Nidacon, Sweeden), the gradient with semen was centrifuged at 300× *g* for 15 min and then washed for 5 min at 300× *g*. Matured oocytes were washed in Fert-TALP [20] medium, transferred to a new dish and inseminated with 1 × 10^6^ spermatozoa/mL. Fertilization was left to occur during 18–20 h, at 38.5 °C, under 5%CO_2_ and high humidity.

### 2.4. Embryo Culture

Presumptive zygotes were denuded from cumulus cells by vortex for 3 min. In each replicate, putative zygotes were divided in two groups according to embryo culture medium (SOF) supplementation: bovine serum albumin (BSA group) or reproductive fluids (RF group). BSA group received supplementation of 3 mg/mL of bovine serum albumin from day 1 to day 8. RF group received supplementation of 1.25% (*v*/*v*) of oviductal fluid (from early luteal phase of the estrous cycle, NaturARTs BOF-EL—Embryocloud, Spain) from day 1 to day 4, and 1.25% (*v*/*v*) of uterine fluid (from mid-luteal phase of the estrous cycle, NaturARTs BUF-ML—Embryocloud, Spain) from day 4 to day 8, as previously described [20]. Putative zygotes were washed twice in the corresponding medium and put into culture in groups of 25 per 25 µL microdrop, covered with parafin oil (Nidoil, Nidacon, Sweden). Incubation conditions were 38.5 °C, 5% CO_2_ and 5% O_2_. On day 4 of culture, all embryos were washed twice in the new corresponding medium and put in a new culture dish.

### 2.5. Embryo Vitrification and Warming

Commercial vitrification media (Kitazato-Dibimed, Spain) and an open-system Cryotop were used following manufacturer’s instructions and as previously described [21]. Embryos on day 7 or 8 of culture and on stage 6 or 7 of development [22] were submitted to vitrification and stored in liquid nitrogen until use. Commercial thawing media (Kitazato-Dibimed, Spain) was used to warm vitrified embryos. Warming was performed according to manufacturer’s instructions and performed less than 4 h before embryo transfer. Embryos were loaded in 0.25 mL straws with commercial medium (BO-Transfer, IVF-Bioscience, Denmark).

### 2.6. Recipient Synchronization and Embryo Transfer

Holstein multiparous dairy cows from a commercial farm (El Barranquillo SL, Spain) were synchronized using double-Ovsynch protocol. Reproductive ultrasound evaluation on the previous day of the transfer was made in order to discard recipients that failed or had delayed ovulation. Recipients were on their day 6, 7 or 8 of the estrus cycle and embryo transfer (ET) was made non-surgically to the ipsilateral uterine horn from ovulation (one embryo per recipient).

### 2.7. Artificial Insemination

Cows were artificially inseminated with frozen-thawed semen from the same Asturian-Valley bull used for IVP. Synchronization was made the same way described earlier (double-Ovsynch protocol) and cows were inseminated on day 0 (presumptive day of estrus).

### 2.8. Pregnancy Detection and Follow-up until Parturition

Pregnancy was detected at day 30 ± 3 of gestation by rectal ultrasonography (Easi-Scan™, BCF Technology, Scotland, UK). Measurement of the crown-rump length (mm) was performed to the conceptuses. Confirmation of pregnancy was repeated at days 60, 90, 150, and 210 of pregnancy. If parturition had not occurred by day 283 ± 2 of gestation, labour was induced with 0,150 mg d-cloprostenol q24h. No C-sections were performed but human intervention was available when calving was difficult. Calving was considered “easy” if little or no help was necessary and “difficult” if heavy assistance was needed. Calves’ weight was assessed between 0 to 4 h after birth, using a weight scale.

### 2.9. Blood Collection and Analysis

Blood from recipient cows was collected via puncture of the median caudal vein with plain tubes (Vacutainer, BD Spain) on the day of ET or 7 days after-estrus for AI group, from here on referred to as “day 7” in both cases. Blood from pregnant recipients was also collected on day 30, 90, 150, and 210. Samples were centrifuged at 1000× *g* for 20 min at room temperature and plasma collected and stored (−20 °C) until analysis. Hormone levels—anti-Müllerian (AMH, ng/mL), estradiol (E2, pg/mL), progesterone (P4, ng/mL) and cortisol (nmol/L)—were measured with ECLIA assay (electrochemiluminescence immunoassay) using a Cobas^®^ e801 system (Roche Diagnostics GmbH, Mannheim, Germany). Five samples of estradiol were not correctly measured and gave values below the detection (<5 ng/mL; a mean value of the previous/following measurement of the individual cow was made and used as a replacement).

### 2.10. Statistical Analysis

For statistical analysis, we followed two different approaches: In the first one, we evaluated the three groups (AI, BSA and RF) independently; in the second one, we compared the data from AI group versus the pooled data of BSA and RF groups, with the intention of getting information about the effect of the ART procedure used (i.e., in vivo fertilized embryos (AI) versus in vitro produced embryos, where IVP = RF + BSA data).

Data were tested for normality using Shapiro–Wilk test (*p* > 0.05) followed by either one-way parametric ANOVA/t-test when approved for normality, or non-parametric Kruskal–Wallis/Mann Whitney U when not normally distributed. Multiple comparisons tests (Tukey/Dunn) were used when significant difference was found (*p* < 0.05). Hormonal parameters were analysed using ANOVA for repeated measures, with Geisser–Greenchouse’s correction applied when data did not follow sphericity. Multiple comparisons tests were performed using Tukey for group analysis and Sidak for type of ART analysis, and *p* < 0.05 was considered significant.

Data presented are mean ± SEM, unless otherwise indicated. The software used was GraphPad Prism version 8.4.0 for Windows (GraphPad Software, San Diego, CA, USA).

## 3. Results

### 3.1. Rate of In Vitro Produced Embryos, Pregnancy Maintenance and Conceptus Size after Embryo Transfer/Artificial Insemination, Were Similar between Groups

Cleavage and blastocyst rate with BSA or RF as supplements to the embryo culture medium was not significantly different (Table 1).

Pregnancy rates were also similar between groups. Table 2 describes the confirmation of pregnancy at different timelines and no statistical difference was found between groups, both in terms of pregnancy rates as well as pregnancy loss.

**Figure 1:**
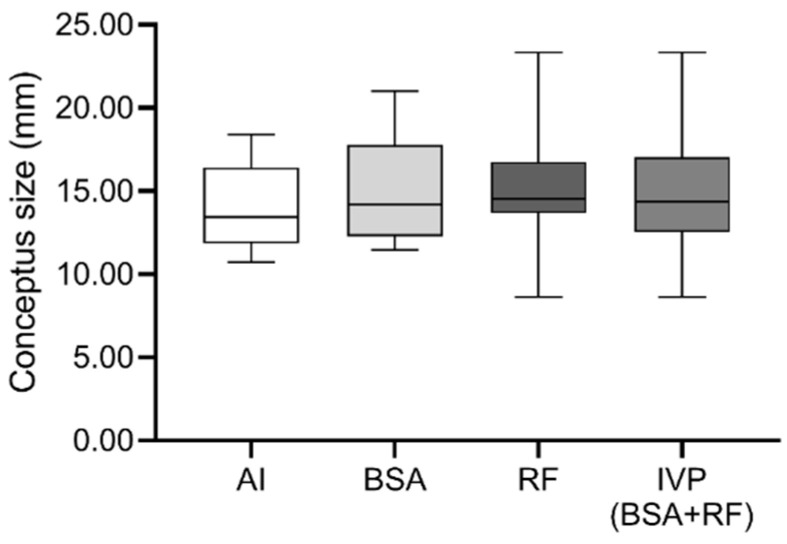
Distribution of conceptus size at day 30 of gestation of embryos produced by artificial insemination (AI), bovine serum albumin (BSA), reproductive fluids (RF), or in vitro-produced (IVP, corresponding of BSA + RF). The boxplot is represented by the first quartile, the median and the third quartile, with minimum and maximum as whiskers.

Conceptus size was also not significantly different between groups (Figure 1).

### 3.2. Recipient’s Hormonal Levels at Day 7, 30, 90, and 210 of Gestation Showed Differences at Specific Time-Points

**Figure 2:**
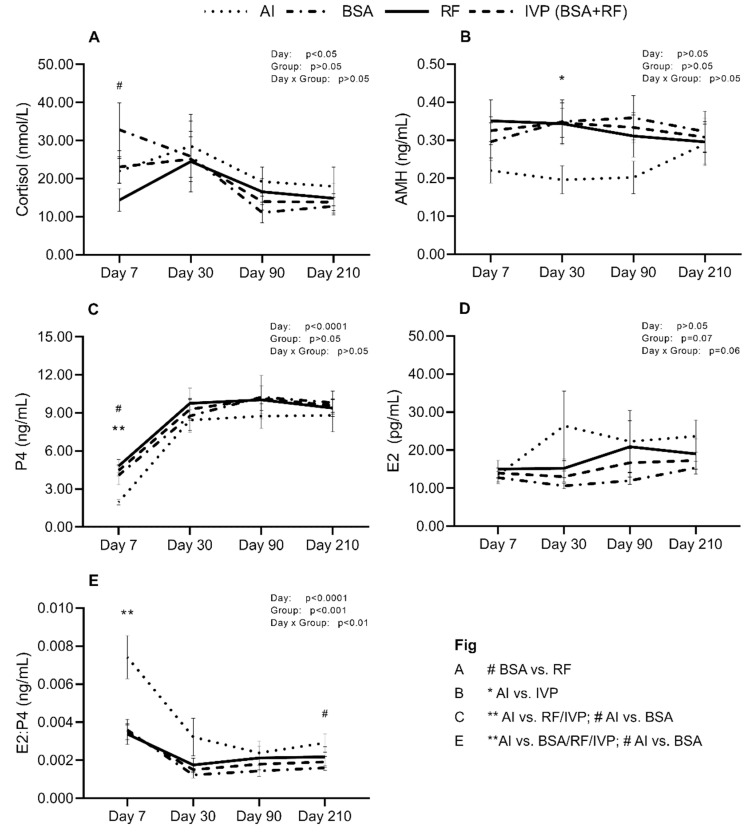
Distribution of mean ± SEM values, of (**A**). Cortisol, (**B**). Anti-Müllerian hormone (AMH), (**C**). Progesterone (P4), (**D**). Estrogen (E2) and (**E**). E2:P4 ratio, in pregnant recipients from artificial insemination (AI) group, bovine serum albumin group (BSA), reproductive fluids group (RF) or in vitro produced (IVP, corresponding of BSA + RF) across gestation time. * *p* < 0.05, ** *p* < 0.01 and # *p* < 0.09.

Figure 2 represents the evolution of hormonal levels through day 7, 30, 90, and 210 of pregnancy; their statistical differences and influence of the day of collection; the group; and day x group interaction. Cortisol levels were significantly affected by the day of collection, and particularly at day 7, showed a tendency to be higher in BSA recipients than RF recipients (Figure 2A, *p* < 0.09). AMH levels of AI recipients showed a significant decrease on day 30 when compared to IVP (Figure 2B, *p* < 0.05). P4 concentrations were influenced by the day of collection, being significantly lower on AI recipients when facing RF or IVP (*p* < 0.01) but just a tendency when compared to BSA (Figure 2C, *p* < 0.09). E2 had a tendency to be influenced by the group or day x group interaction, but no other significant difference was shown (Figure 2D). E2/P4 ratio was highly variable due to the day, group and day x group interaction, having AI recipients higher mean levels on day 7 vs. BSA/RF/IVP (*p* < 0.01) and a tendency on day 210 vs. BSA (Figure 2E, *p* < 0.09).

### 3.3. Gestation Length, Parturitions and Neonatal Period

Mean gestation length, minimum and maximum length for the non-induced parturitions is shown in Table 3.

One premature calf was delivered naturally with 240 days and another recipient, due to productivity reasons, was not induced and the gestation lasted 298 days. Induced parturitions were necessary in some cases for BSA and RF groups (Table 4), mostly due to the fear of increased birth weight and consequently calving difficulties, and resulted in a higher tendency of IVP vs. AI of induced parturitions (*p* = 0.0573). Calving ease showed no statistical differences between groups. Percentage of male calves was high in absolute values, but without statistical differences between groups.

**Figure 3:**
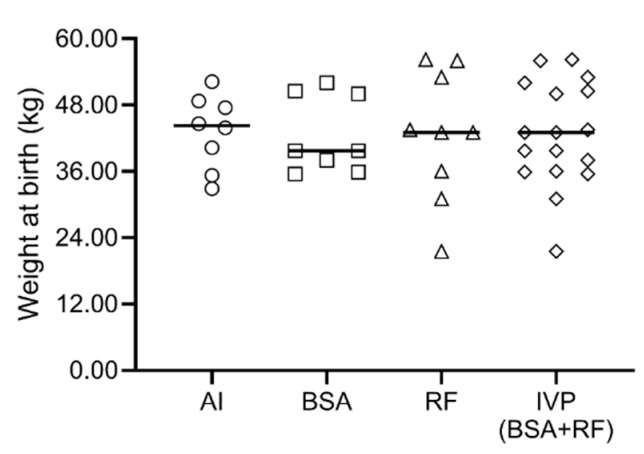
Weight at birth of calves born through artificial Insemination (AI) group, bovine serum albumin group (BSA), reproductive fluids group (RF) or in vitro-produced (IVP, corresponding of BSA + RF). The line represents the median and each symbol represents one animal.

Weight at birth was also not different between groups (Figure 3).

Neonatal mortality happened in one calf for BSA group and in four calves for RF group and statistically did not show any difference between groups (Table 4), but a tendency of higher mortality in RF vs. AI happened. Details on age at death, sex, birth weight, and cause of death are shown in Table 5.

## 4. Discussion

The in vitro embryo production in cattle industry has been drastically increasing over the past few years. Today, it is necessary not only to optimize its final yield, but also to assure that we are producing high quality and healthy animals and that calving problems are minimum. To this end, culture conditions and recipient selection and monitoring appear as main points to keep under control. Culture conditions of IVP are still in need of improvement, not only in cattle but in mammals in general [10,23,24]. The pre-implantation embryo represents a critical stage where all environmental conditions might reach higher relevance than at any other stage. As reviewed by Vajta et al. [10], current culture conditions, ranging from media composition to temperature of incubation, are not a unanimity, suggesting that there is still a lot of unknown factors in the biological environment that need to be studied and addressed. The addition of RF, such as oviductal and uterine fluids, has been proposed before [12,25] as a potential tool to improve the embryo quality. Hamdi et al. [12], using both fluids as supplements to embryo culture media, obtained similar blastocyst rates to those reached with fetal calf serum or even BSA supplementation. Despite not showing any improvement of blastocyst yield, embryos produced with RF supplementation showed higher survivability after vitrification-warming than serum-derived embryos, a downregulation of genes related with oxidative stress in comparison with BSA-derived embryos as well as significantly lower reactive oxygen species when compared to both serum and BSA groups. However, no data related to the ability of these embryos to implant or to develop to term after being transferred into recipient cows had been published until now, thus our work is the first producing live birth calves from IVP embryos cultured with reproductive fluids as supplements.

Pregnancy rates from vitrified-warmed IVP embryos are known to be inferior when compared to fresh IVP embryo transfers [5,26,27], with very few studies showing equal values [28]. Our results showed similar values at the first diagnosis for both BSA and RF groups, and these rates were slightly lower when compared to some studies that used abattoir-derived oocytes in IVP plus vitrification [5,26,29], but within the range or even higher than others that used slow freezing method [6,30]. Our AI pregnancy rates were lower than expected, probably explained by the high temperatures during the season in southern Spain, which induces heat-stress to the cows. Pregnancy loss was not significantly different between groups, and the percentage of lost pregnancies is in accordance to another study [5] that used vitrified embryos and had 25.9% pregnancy loss between diagnoses and calving.

Progesterone, being a key hormone associated with uterus preparation to receive the embryo, is one of the most studied hormones in cattle. According to Stronge et al. [31], low levels of P4 during the first 5 days after estrus are associated with low fertility. In our data, we found the normal physiological response that is the increase of P4 between day 7 and day 30. However, between the pregnant animals of our experimental groups, lower levels of P4 in AI recipients were found when compared to RF group or even IVP. This difference might be related to the fact that IVP recipients were chosen carefully, with evaluation of corpus luteum on the day prior to ET, and non-conforming recipients were discarded. On the contrary, AI recipients were inseminated after synchronization, without later confirmation of ovulation. It could be that AI recipients, even though pregnancy was achieved, may have ovulated later than expected in comparison to IVP recipients and consequently, the P4 levels were lower. Nevertheless, both our AI and IVP groups had P4 values that are within the range of normality for day 7 [32,33,34,35,36]. After this difference on day 7, by day 30 and onwards, P4 concentrations in all groups were similar. This could also help to explain why we did not find any differences regarding the conceptus size. Higher levels of circulating P4 have been associated with conceptus elongation as early as day 14 [36,37]. Supplementation of progesterone in early days post-estrus has also been responsible for higher conceptus length [38,39]. Although day 7 P4 concentrations from our groups differ significantly between AI recipients and RF/IVP recipients, the fact that by day 30 P4 levels were similar, might have led to non-existing differences in size of conceptus. Whether the rise of P4 concentrations on AI group between day 7 and day 30 was sufficient to catch up with IVP groups or if the possible higher embryo quality (and interferon-τ secretion) from AI group were responsible for this lack of difference, remains to be explained.

The role of cortisol during pregnancy is still not totally understood. It is known that glucocorticoids have roles in many physiological processes (reviewed by [40,41]), but its importance on modulating the uterine environment and consequently, influencing embryo implantation, has been the subject of attention. In cattle, interferon-τ is part of the maternal-recognition system and it is secreted by the trophoblast into the uterus especially between day 13–21 of pregnancy, and as so, Majewska et al. [40] correlated the relationship between cortisol and interferon-τ. This study, after using epithelial and stromal cells from pregnant and non-pregnant cows, concluded that interferon-τ modulates the conversion of cortisone to cortisol just after 12 h of incubation, but that response is also dose-dependent, meaning that there is a necessary dose of interferon-τ to achieve this modulation. This is an important step, as by increasing cortisol levels in vivo, the prostaglandins will be down-regulated and the cow will be able to maintain the corpus luteum active. In our study, the day of collection showed a high influence over the results. Mean levels of cortisol by day 30 were very similar between groups, and then, in general, showed a decrease during the rest of the pregnancy. The only non-conforming group was BSA recipients that showed really high levels at day 7, then a decrease on day 30 continuing until day 90, and finishing with a slight increase at day 210. However, this response did not influence, as far as we understand, the outcomes of the pregnancies. Whether the vitrified-warmed IVP embryos differ from those produced in vivo in the expression of interferon-τ is not yet clear (reviewed by [42]).

Anti-Müllerian hormone is a growth factor produced by granulosa cells that has been positively correlated with pregnancy rate and maintenance, among other traits [43,44]. This hormone might be used as a biomarker for fertility, and even though its value varies much between individuals and breeds [43], it is quite stable within one individual and does not vary much within the estrus cycle. In general, our AI recipients showed lower levels of AMH when compared to the other groups, but this difference was only significant when comparing to IVP data on day 30. All the obtained values are in the mean average range of AMH concentration in Holstein cows [43], which is around 0.250 ng/mL, and could be considered an intermediate value (not too high nor too low). This is important because the same study [43] unveiled that cows with low AMH had greater risk for early pregnancy loss, which was not the case of our AI recipients. Nevertheless, these studies need to be taken into careful consideration, since they only use AI as a pregnancy method, and we are not fully confident that the embryo does not itself have an influence over their mother’s hormonal values, as it happens with foetal sex [45].

Oestrogen levels were quite similar within groups of pregnant cows. However, when evaluating levels of E2, it is important to compare with P4 levels, thus the necessity of E2:P4 ratio. This ratio showed a high influence of the day, group and day x group interaction, and much higher levels for AI recipients vs. BSA/RF/IVP on day 7, as expected after the differences found on P4 levels. Later on, at day 210 there was again a tendency of difference between AI and BSA recipients. With the exception of day 7 differences, where the reason for the higher E2:P4 was the low P4 concentrations, the day 210 tendency was due to high levels of E2. Fuchs et al. [46] described that concentrations of E2 tend to decrease after day 20 of estrus until day 250 where they start to rise again to prepare for parturition. Nonetheless, they also describe E2 concentrations at day 20 around 24 pg/mL, which is within the range for our AI group, meaning that it is not that AI recipients have high levels of E2, but rather that BSA cows expressed lower levels than expected. However, all these eight BSA recipients gave birth to live calves.

Gestation lengths are reported to be longer for IVP pregnancies in some studies [15,47,48,49,50,51,52], but similar to AI in others [5,53]. It should be noted that the percentage of induced parturitions was higher in IVP (regardless of the RF or BSA supplementation) pregnancies than AI pregnancies (with a tendency to be significantly different), and those pregnancies lengths (*n* = 7) were not accounted for mean gestation length, which if it did, would probably contribute to an increase in the mean gestation length. Moreover, one of the RF-pregnancies was not induced and was led to term without induction, lasting 298 days. Although gestation length is considered a highly heritable trait (reviewed by [54]), it is important to refer to the fact that the same bull was used in both AI and IVP groups. The mean value for gestation length for calves from Asturian Valley breed is 286.6 days for female and 287.5 days for male calves [55], but since AI cows were Holstein and there is no traceability on the female donors for IVP embryos (crossbred beef), it is not possible to attribute these differences to any of the estimated breed values.

Parturitions in IVP-derived pregnancies are known to have a tendency to be more difficult than AI pregnancies [29,51,52,56]. In our study, three IVP pregnancies were considered difficult while all AI calving were evaluated as easy, not being statistically relevant. The proportion of male calves being higher in IVP vs. AI has also been pointed as a distinct characteristic [15]. In fact, some studies reported higher percentage of male calves [3,50,56,57], while on the contrary others reported a similar proportion [58,59,60]. Nevertheless, our results were that AI group had the highest proportion of males vs. female, followed by BSA group, and the lowest proportion was given by the RF group.

Weight at birth was not statistically different between groups. This is in disagreement with previous studies that reported higher birth weight for IVP calves [30,47,48,49,50,52,53,57,59,61,62], but in agreement with others [63]. Interestingly, our heaviest calf was IVP-derived (56.2 kg) as well as our lightest (21.5 kg). The AI *Asturian x Holstein* combination brought a high proportion of calves with weights within 40–50 kg.

Mortality during the neonatal period happened only with IVP calves. Numabe et al. [64], Behboodi et al. [57] and Van Wagtendonk-De Leeuw et al. [53] reported higher incidence of perinatal mortality in IVP-derived calves than AI-derived calves. Both calves that died during parturition were heavy calves (>50 kg), which is an attribute given to the IVP-origin. The calf that died 2 days after birth was in agreement with Jenkins et al. [54] that associated longer gestation to higher perinatal mortality. Another calf died after surviving 12 days of premature deliver (240 days). Finally, the last calf that died at 13 days old was due to one of the most common causes of death in neonatal calves, diarrhoea.

## 5. Conclusions

In this study we applied new strategies in ART towards the more physiological approach of IVP, by including natural reproductive fluids in the culture media. With our limited sample size, we were not able to find worthy differences at the first stages of embryo development, implantation and parturitions, derived from the addition of such fluids. By contrast, we found that most of the parameters studied were similar between both types of IVP derived embryos and the in vivo-derived embryos, suggesting the IVP technology used was efficient enough for the safe production of calves. Since there are changes that may only be detected phenotypically later in life, as one may understand from other works [53], it is imperative to study the development and growth of these animals in order to reach more consistent conclusions. Moreover, it is important to assess if the changes that have been previously found in pre-implantation stages are in any form present in the live animals, particularly regarding large offspring syndrome. The fact that these ART are being used globally to improve genetics of the herd or even to overcome heat stress in affected areas, make them essential tools that need to be studied thoroughly.

## Figures and Tables

**Table 1:** Total presumptive zygotes, cleavage and blastocyst rate of in vitro produced embryos bovine serum albumin (BSA) or reproductive fluids (RF).

Group	Total Presumptive Zygotes	Cleavage Rate		Blastocyst Yield	
	*n*	*%*	*n*	*%*	*n*
BSA	360	85.6 ± 1.9	308	26.7 ± 2.3	96
RF	429	85.6 ± 1.7	367	25.9 ± 2.1	111

Percentages are shown as mean ± SEM.

**Table 2:** Pregnancy confirmation during gestation and parturitions of artificial insemination (AI), bovine serum albumin (BSA), reproductive fluids (RF) and IVP (BSA and RF groups data combined) groups.

Group	Recipients	Day 30		Day 60		Day 90		Day 150		Day 210		Parturition	
	*n*	*%*	*n*	*%*	*n*	*%*	*n*	*%*	*n*	*%*	*n*	*%*	*n*
AI	35	22.9	8	22.9	8	22.9	8	22.9	8	22.9	8	22.9	8
BSA	45	22.2	10	17.8	8	17.8	8	17.8	8	17.8	8	17.8	8
RF	54	22.2	12	18.5	10	18.5	10	16.7	9	16.7	9	16.7	9
IVP (BSA + RF)	99	22.2	22	18.2	18	18.2	18	17.3	17	17.3	17	17.3	17

Percentages are means and n are number of animals.

**Table 3:** Gestation length of non-induced parturitions from pregnant cows of artificial insemination (AI), bovine serum albumin (BSA), reproductive fluids (RF) or in vitro-produced (IVP, corresponding of BSA + RF).

Group	*n*	Gestation Length	Minimum	Maximum
AI	8	281.1 ± 0.7	277	284
BSA	5	280.4 ± 1.4	275	282
RF	5	273.6 ± 9.7	240	298 *
IVP (BSA + RF)	10	277.0 ± 4.8	240	298 *

Gestation length is represented as mean ± SEM; * this parturition was not induced by decision of the farmers.

**Table 4:** Induced parturitions, calving ease score, male calves, and neonatal mortality within groups of artificial insemination (AI), bovine serum albumin (BSA), reproductive fluids (RF) or in vitro produced (IVP, corresponding of BSA + RF).

Group	Parturitions	Induced Parturition		Calving Ease				Male Calves		Neonatal Mortality	
				Easy		Difficult					
	*n*	%	*n*	%	*n*	%	*n*	%	*n*	%	*n*
AI	8	0 ^†^	0	100	8	0	0	75.0	6	0 *	0
BSA	8	37.5	3	87.5	7	12.5	1	62.5	5	12.5	1
RF	9	44.4	4	77.8	7	22.2	2	55.6	5	44.4 *	4
IVP (BSA+RF)	17	41.2 ^†^	7	82.4	14	17.6	3	58.8	10	29.4	5

Calving ease was scored as easy if it required little to no assistance or difficult if it needed moderate to heavy assistance (i.e., surgery/veterinarian intervention). ^†^ AI vs. IVP *p* = 0.0573; * AI vs. RF *p* = 0.0752.

**Table 5:** Cases of neonatal mortality from calves born by embryo transfer of vitrified-warmed embryos produced with reproductive fluids (RF) or bovine serum albumin (BSA) as supplements to culture medium.

Characteristic	Case 1	Case 2	Case 3	Case 4	Case 5
Age at death (days)	12	0	2	13	0
Sex	Female	Male	Female	Male	Female
Birth weight (kg)	21.5	56.0	43.0	52.0	56.2
Group	RF	RF	RF	BSA	RF
Cause of death	Premature calf ^ND^	Dystocia	Septicaemia	Diarrhoea	Dystocia, spine fracture

^ND^ Stands for not determined.

## References

[1] Viana J. (2019). IETS Data Retrieval Committee. 2018 statistics of embryo production and transfer in domestic farm animals. Embryo Technol. Newsl..

[2] Drost M., Ambrose J.D., Thatcher M.-J., Cantrell C.K., Wolfsdorf K.E., Hasler J.F., Thatcher W.W. (1999). Conception rates after artificial insemination or embryo transfer in lactating dairy cows during summer in florida. Theriogenology.

[3] Pontes J.H.F., Nonato-Junior I., Sanches B.V., Ereno-Junior J.C., Uvo S., Barreiros T.R.R., Oliveira J.A., Hasler J.F., Seneda M.M. (2009). Comparison of embryo yield and pregnancy rate between in vivo and in vitro methods in the same Nelore (*Bos indicus*) donor cows. Theriogenology.

[4] Taverne M., Breukelman S., Perényi Z., Dieleman S., Vos P., Jonker H., de Ruigh L., van Wagtendonk-de Leeuw J.M., Beckers J.-F. (2002). The monitoring of bovine pregnancies derived from transfer of in vitro produced embryos. Reprod. Nutr. Dev..

[5] Stewart B.M., Block J., Morelli P., Navarette A.E., Amstalden M., Bonilla L., Hansen P.J., Bilby T.R. (2011). Efficacy of embryo transfer in lactating dairy cows during summer using fresh or vitrified embryos produced in vitro with sex-sorted semen. J. Dairy Sci..

[6] Dochi O., Takahashi K., Hirai T., Hayakawa H., Tanisawa M., Yamamoto Y., Koyama H. (2008). The use of embryo transfer to produce pregnancies in repeat-breeding dairy cattle. Theriogenology.

[7] Holm P., Booth P.J., Callesen H. (2002). Kinetics of early in vitro development of bovine in vivo-and in vitro-derived zygotes produced and/or cultured in chemically defined or serum-containing media. Reproduction.

[8] Rizos D., Clemente M., Bermejo-Alvarez P., De La Fuente J., Lonergan P., Gutiérrez-Adán A. (2008). Consequences of in vitro culture conditions on embryo development and quality. Reprod. Domest. Anim..

[9] Sudano M.J., Paschoal D.M., da Silva Rascado T., Magalhães L.C.O., Crocomo L.F., de Lima-Neto J.F., da Cruz Landim-Alvarenga F. (2011). Lipid content and apoptosis of in vitro-produced bovine embryos as determinants of susceptibility to vitrification. Theriogenology.

[10] Vajta G., Rienzi L., Cobo A., Yovich J. (2010). Embryo culture: Can we perform better than nature?. Reprod. Biomed. Online.

[11] Wydooghe E., Heras S., Dewulf J., Piepers S., Van Den Abbeel E., De Sutter P., Vandaele L., Van Soom A. (2014). Replacing serum in culture medium with albumin and insulin, transferrin and selenium is the key to successful bovine embryo development in individual culture. Reprod. Fertil. Dev..

[12] Hamdi M., Lopera-vasquez R., Maillo V., Sanchez-Calabuig M.J., Núnez C., Gutierrez-Adan A., Rizos D. (2018). Bovine oviductal and uterine fluid support in vitro embryo development. Reprod. Fertil. Dev..

[13] García-Martínez S., Sánchez Hurtado M.A., Gutiérrez H., Sánchez Margallo F.M., Romar R., Latorre R., Coy P., López Albors O. (2018). Mimicking physiological O_2_ tension in the female reproductive tract improves assisted reproduction outcomes in pig. Mol. Hum. Reprod..

[14] Ferraz M., Henning H., Costa P.F., Malda J., Melchels F.P., Wubbolts R., Stout T.A.E., Vos P.L.A.M., Gadella B.M. (2017). Improved bovine embryo production in an oviduct-on-a-chip system: Prevention of poly-spermic fertilization and parthenogenic activation. Lab Chip.

[15] Hasler J.F. (2000). In-vitro production of cattle embryos: Problems with pregnancies and parturition. Hum. Reprod..

[16] Paschoal D.M., Sudano M.J., Schwarz K.R.L., Maziero R.R.D., Guastali M.D., Crocomo L.F., Magalhães L.C.O., Martins A., Leal C.L.V., da Cruz Landim-Alvarenga F. (2017). Cell apoptosis and lipid content of in vitro–produced, vitrified bovine embryos treated with forskolin. Theriogenology.

[17] Rizos D., Ward F., Duffy P., Boland M.P., Lonergan P. (2002). Consequences of bovine oocyte maturation, fertilization or early embryo development in vitro versus in vivo: Implications for blastocyst yield and blastocyst quality. Mol. Reprod. Dev..

[18] Ferraz P.A., Burnley C., Karanja J., Viera-Neto A., Santos J.E.P., Chebel R.C., Galvão K.N. (2016). Factors affecting the success of a large embryo transfer program in Holstein cattle in a commercial herd in the southeast region of the United States. Theriogenology.

[19] Sanches B.V., Lunardelli P.A., Tannura J.H., Cardoso B.L., Colombo Pereira M.H., Gaitkoski D., Basso A.C., Arnold D.R., Seneda M.M. (2016). A new direct transfer protocol for cryopreserved IVF embryos. Theriogenology.

[20] Parrish J.J. (2014). Bovine in vitro fertilization: In vitro oocyte maturation and sperm capacitation with heparin. Theriogenology.

[21] Lopes J.S., Canha-Gouveia A., París-Oller E., Coy P. (2019). Supplementation of bovine follicular fluid during in vitro maturation increases oocyte cumulus expansion, blastocyst developmental kinetics, and blastocyst cell number. Theriogenology.

[22] Bó G.A., Mapletoft R.J. (2013). Evaluation and classification of bovine embryos. Anim. Reprod..

[23] Lonergan P., Fair T. (2014). The ART of studying early embryo development: Progress and challenges in ruminant embryo culture. Theriogenology.

[24] Sunde A., Brison D., Dumoulin J., Harper J., Lundin K., Magli M.C., Van den Abbeel E., Veiga A. (2016). Time to take human embryo culture seriously. Hum. Reprod..

[25] Canovas S., Ivanova E., Romar R., García-Martínez S., Soriano-Úbeda C., García-Vázquez F.A., Saadeh H., Andrews S., Kelsey G., Coy P. (2017). DNA methylation and gene expression changes derived from assisted reproductive technologies can be decreased by reproductive fluids. eLife.

[26] Block J., Bonilla L., Hansen P.J. (2010). Efficacy of in vitro embryo transfer in lactating dairy cows using fresh or vitrified embryos produced in a novel embryo culture medium. J. Dairy Sci..

[27] Chebel R.C., Demétrio D.G.B., Metzger J. (2008). Factors affecting success of embryo collection and transfer in large dairy herds. Theriogenology.

[28] Do V.H., Catt S., Amaya G., Batsiokis M., Walton S., Taylor-Robinson A.W. (2018). Comparison of pregnancy in cattle when non-vitrified and vitrified in vitro-derived embryos are transferred into recipients. Theriogenology.

[29] Bonilla L., Block J., Denicol A.C., Hansen P.J. (2014). Consequences of transfer of an in vitro-produced embryo for the dam and resultant calf. J. Dairy Sci..

[30] Lazzari G., Wrenzycki C., Herrmann D., Duchi R., Kruip T., Niemann H., Galli C. (2002). Cellular and molecular deviations in bovine in vitro-produced embryos are related to the large offspring syndrome. Biol. Reprod..

[31] Stronge A.J.H., Sreenan J.M., Diskin M.G., Mee J.F., Kenny D.A., Morris D.G. (2005). Post-insemination milk progesterone concentration and embryo survival in dairy cows. Theriogenology.

[32] Schrick F.N., Inskeep E.K., Butcher R.L. (1993). Pregnancy rates for embryos transferred from early postpartum beef cows into recipients with normal estrous cycles. Biol. Reprod..

[33] Silva J.C., Costa L.L., Silva J.R. (2002). Plasma progesterone profiles and factors affecting embryo-fetal mortality following embryo transfer in dairy cattle. Theriogenology.

[34] Breukelman S.P., Perényi Z., Taverne M.A.M., Jonker H., van der Weijden G.C., Vos P.L.A.M., de Ruigh L., Dieleman S.J., Beckers J.F., Szenci O. (2012). Characterisation of pregnancy losses after embryo transfer by measuring plasma progesterone and bovine pregnancy-associated glycoprotein-1 concentrations. Vet. J..

[35] Shorten P.R., Ledgard A.M., Donnison M., Pfeffer P.L., McDonald R.M., Berg D.K. (2018). A mathematical model of the interaction between bovine blastocyst developmental stage and progesterone-stimulated uterine factors on differential embryonic development observed on Day 15 of gestation. J. Dairy Sci..

[36] O’Hara L., Scully S., Maillo V., Kelly A.K., Duffy P., Carter F., Forde N., Rizos D., Lonergan P. (2012). Effect of follicular aspiration just before ovulation on corpus luteum characteristics, circulating progesterone concentrations and uterine receptivity in single-ovulating and superstimulated heifers. Reproduction.

[37] Spencer T.E., Forde N., Lonergan P. (2016). The role of progesterone and conceptus-derived factors in uterine biology during early pregnancy in ruminants. J. Dairy Sci..

[38] O’Hara L., Forde N., Kelly A.K., Lonergan P. (2014). Effect of bovine blastocyst size at embryo transfer on day 7 on conceptus length on day 14: Can supplementary progesterone rescue small embryos?. Theriogenology.

[39] Clemente M., de La Fuente J., Fair T., Al Naib A., Gutierrez-Adan A., Roche J.F., Rizos D., Lonergan P. (2009). Progesterone and conceptus elongation in cattle: A direct effect on the embryo or an indirect effect via the endometrium?. Reproduction.

[40] Majewska M., Lee H.Y., Tasaki Y., Acosta T.J., Szostek A.Z., Siemieniuch M., Okuda K., Skarzynski D.J. (2012). Is cortisol a modulator of interferon tau action in the endometrium during early pregnancy in cattle?. J. Reprod. Immunol..

[41] Michael A.E., Papageorghiou A.T. (2008). Potential significance of physiological and pharmacological glucocorticoids in early pregnancy. Hum. Reprod. Update.

[42] Ealy A.D., Wooldridge L.K., Mccoski S.R. (2019). Post-transfer consequences of in vitro-produced embryos in cattle. J. Anim. Sci..

[43] Ribeiro E.S., Bisinotto R.S., Lima F.S., Greco L.F., Morrison A., Kumar A., Thatcher W.W., Santos J.E.P. (2014). Plasma anti-Müllerian hormone in adult dairy cows and associations with fertility. J. Dairy Sci..

[44] Nawaz M.Y., Jimenez-Krassel F., Steibel J.P., Lu Y., Baktula A., Vukasinovic N., Neuder L., Ireland J.L.H., Ireland J.J., Tempelman R.J. (2018). Genomic heritability and genome-wide association analysis of anti-Müllerian hormone in Holstein dairy heifers. J. Dairy Sci..

[45] Stojsin-Carter A., Costa N.N., De Morais R., De Bem T.H., Costa M.P., Carter T.F., Gillis D.J., Neal M.S., Ohashi O.M., Miranda M.S. (2017). Fetal sex alters maternal anti-Mullerian hormone during pregnancy in cattle. Anim. Reprod. Sci..

[46] Fuchs A.-R., Helmer H., Behrens O., Liu H.-C., Antonian L., Chang S.M., Fields M.J. (2005). Oxytocin and Bovine Parturition: A Steep Rise in Endometrial Oxytocin Receptors Precedes Onset of Labor1. Biol. Reprod..

[47] Kruip T.A.M., Den Daas J.H.G. (1997). In vitro produced and cloned embryos: Effects on pregnancy, parturition and offspring. Theriogenology.

[48] Sinclair K.D., Broadbent P.J., Dolman D.F. (1995). In vitro produced embryos as a means of achieving pregnancy and improving productivity in beef cows. Anim. Sci..

[49] Yang B., Im G., Park S. (2001). Characteristics of Korean native, Hanwoo, calves produced by transfer of in vitro produced embryos. Anim. Reprod. Sci..

[50] Park Y.-S., Kim S.-S., Kim J.-M., Park H.-D., Byun M.-D. (2005). The effects of duration of in vitro maturation of bovine oocytes on subsequent development, quality and transfer of embryos. Theriogenology.

[51] Pimenta-Oliveira A., Oliveira-Filho J.P., Dias A., Gonçalves R.C. (2011). Morbidity-mortality and performance evaluation of Brahman calves from in vitro embryo production. BMC Vet. Res..

[52] Van Wagtendonk-De Leeuw A.M., Aerts B.J.G., Den Daas J.H.G. (1998). Abnormal offspring following in vitro production of bovine preimplantation embryos: A field study. Theriogenology.

[53] Siqueira L.G.B., Dikmen S., Ortega M.S., Hansen P.J. (2017). Postnatal phenotype of dairy cows is altered by in vitro embryo production using reverse X-sorted semen. J. Dairy Sci..

[54] Jenkins G.M., Amer P., Stachowicz K., Meier S. (2016). Phenotypic associations between gestation length and production, fertility, survival, and calf traits. J. Dairy Sci..

[55] Goyache F., Fernandez I., Alvarez I., Royo L.J., Gutierrez J.P. (2002). Gestation length in the asturiana de los valles beef cattle breed and its relationship with birth weight and calving ease. Arch. Zootec..

[56] Van Wagtendonk-de Leeuw A.M., Mullaart E., de Roos A.P.W., Merton J.S., den Daas J.H.G., Kemp B., de Ruigh L. (2000). Effects of different reproduction techniques: AI, moet or IVP, on health and welfare of bovine offspring. Theriogenology.

[57] Behboodi E., Anderson G.B., BonDurant R.H., Cargill S.L., Kreuscher B.R., Medrano J.F., Murray J.D. (1995). Birth of large calves that developed from in vitro-derived bovine embryos. Theriogenology.

[58] Schmidt M., Greve T., Avery B., Beckers J.F., Sulon J., Hansen H.B. (1996). Pregnancies, calves and calf viability after transfer of in vitro produced bovine embryos. Theriogenology.

[59] Jacobsen H., Schmidt M., Holm P., Sangild P.T., Vajta G., Greve T., Callesen H. (2000). Body dimensions and birth and organ weights of calves derived from in vitro produced embryos cultured with or without serum and oviduct epithelium cells. Theriogenology.

[60] Merton J.S., Knijn H.M., Flapper H., Dotinga F., Roelen B.A.J., Vos P.L.A.M., Mullaart E. (2013). Cysteamine supplementation during invitro maturation of slaughterhouse-and opu-derived bovine oocytes improves embryonic development without affecting cryotolerance, pregnancy rate, and calf characteristics. Theriogenology.

[61] Hasler J.F., Henderson W.B., Hurtgen P.J., Jin Z.Q., McCauley A.D., Mower S.A., Neely B., Shuey L.S., Stokes J.E., Trimmer S.A. (1995). Production, freezing and transfer of bovine IVF embryos and subsequent calving results. Theriogenology.

[62] McEvoy T.G., Sinclair K.D., Broadbent P.J., Goodhand K.L., Robinson J.J. (1998). Post-natal growth and development of Simmental calves derived from in vivo or in vitro embryos. Reprod. Fertil. Dev..

[63] Chavatte-Palmer P., Heyman Y., Richard C., Monget P., LeBourhis D., Kann G., Chilliard Y., Vignon X., Renard J.P. (2002). Clinical, hormonal, and hematologic characteristics of bovine calves derived FROM nuclei from somatic cells. Biol. Reprod..

[64] Numabe T., Oikawa T., Kikuchi T., Horiuchi T. (2000). Production efficiency of Japanese black calves by transfer of bovine embryos produced in vitro. Theriogenology.

